# Uromodulin and Tryptophan Metabolite Clearance in Hemodialyzed Patients

**DOI:** 10.3390/jcm15103743

**Published:** 2026-05-13

**Authors:** Izabela Zakrocka, Małgorzata Kozioł, Marta Więckowska-Deroń, Sylwia Boczkowska, Renata Kloc, Tomasz Kocki, Alina Olender, Ewa M. Urbańska, Wojciech Załuska, Andreas Kronbichler

**Affiliations:** 1 Department of Nephrology, Medical University of Lublin, 20-090 Lublin, Poland; wojciech.zaluska@umlub.edu.pl; 2 Department of Medical Microbiology, Medical University of Lublin, 20-093 Lublin, Poland; malgorzata.koziol@umlub.edu.pl (M.K.); marta.wieckowska-deron@umlub.edu.pl (M.W.-D.); alina.olender@umlub.edu.pl (A.O.); 3 Department of Holistic Care and Management in Nursing, Medical University of Lublin, 20-081 Lublin, Poland; sylwia.boczkowska@umlub.edu.pl; 4 Department of Experimental and Clinical Pharmacology, Medical University of Lublin, 20-090 Lublin, Poland; renata.kloc@umlub.edu.pl (R.K.); ewa.urbanska@umlub.edu.pl (E.M.U.); 5 Institute of Health Sciences, The John Paul II Catholic University of Lublin, Konstantynów 1F Street, 20-708 Lublin, Poland; tomasz.kocki@kul.pl; 6 Department of Internal Medicine IV, Nephrology and Hypertension, Medical University Innsbruck, 6020 Innsbruck, Austria

**Keywords:** tryptophan, kynurenine, kynurenic acid, chronic kidney disease, hemodialysis, hemodiafiltration, uremic toxins, uromodulin, kidney tubules, residual kidney function

## Abstract

**Background**: Certain metabolites of the tryptophan-kynurenine (Trp-KYN) pathway, which are primarily cleared via tubular transport, have been linked to end-stage kidney disease (ESKD). Uromodulin—a protein expressed exclusively in the kidneys—is a key regulator of renal structure and function, as well as a direct marker of tubular health. This preliminary study explores the hypothesis that serum uromodulin correlates with Trp-KYN metabolites, potentially revealing new pathophysiological pathways in patients undergoing kidney replacement therapy (KRT). Given the link between serum uromodulin, Trp-KYN metabolites, and tubular function, we examined their correlation in KRT patients. Furthermore, we assessed how various clinical and dialysis parameters influence serum uromodulin levels. **Methods**: A total of 64 stable patients from a single dialysis center receiving hemodialysis (HD) or hemodiafiltration (HDF) were enrolled. Pre- and post-dialysis concentrations of uromodulin, Trp, KYN, kynurenic acid (KYNA), 3-hydroxykynurenine (3-OHKYN), and their reduction ratios (RRs) were established. High-performance liquid chromatography (HPLC) was used to estimate the KYN pathway metabolite levels, whereas uromodulin concentration was measured using an immunoenzymatic assay. **Results**: Detectable serum uromodulin was found in only 30 patients. This group was predominantly male (*p* < 0.001) and characterized by shorter dialysis vintage (*p* < 0.001), a higher prevalence of residual kidney function (RKF) (*p* = 0.001) and diabetes mellitus (*p* = 0.028), higher pre-dialysis serum phosphorus levels (*p* = 0.015), and more frequent use of loop diuretics (*p* = 0.004). Furthermore, univariate analysis revealed significantly higher pre-dialysis (*p* = 0.004) and post-dialysis (*p* = 0.025) serum Trp concentrations in the uromodulin-positive group. Pre-dialysis serum uromodulin concentration correlated positively with pre-dialysis Trp level (*p* < 0.001) and negatively with the pre-dialysis KYN/Trp ratio (*p* = 0.008), but not with other metabolites that are also subject to tubular transport mechanisms. Post-dialysis uromodulin levels correlated positively only with post-dialysis Trp level (*p* = 0.005). Patients treated with HDF had significantly higher RR for uromodulin than those treated with HD (*p* = 0.01). **Conclusions**: The presented data indicate that serum uromodulin levels are correlated with RKF. Additionally, the presence of detectable serum uromodulin may indicate reduced immunological activation, leading to diminished activity within the Trp-KYN pathway.

## 1. Introduction

Chronic kidney disease (CKD) remains one of the most common non-communicable disorders with long-term impact on quality of life and overall survival. Risk of kidney failure and its consequences seem to be independent of the cause of kidney damage. Hence, universal kidney preservation strategies require closer attention. Residual kidney function (RKF) in patients with kidney failure is a well-known predictor of their survival. Given the elevated risk of cardiovascular and all-cause mortality among kidney replacement therapy (KRT) patients, modifying therapeutic approaches is essential to enhance long-term survival. Evidence suggests that hemodiafiltration (HDF) provides superior clinical outcomes to hemodialysis (HD) [1]. Nevertheless, certain drawbacks persist, including the possibility of greater amino acid depletion during the procedure [2].

Uromodulin is a protein exclusively expressed in the kidney, mainly in the thick ascending loop of Henle (TAL) [3], in the early distal tubule regions [4], and glomeruli tuft [5]. It is considered a key regulator of kidney structure and function. A majority of studies concern urinary excretion of uromodulin, and its potential contribution to urinary tract infections and nephrocalcinosis [6]. However, recent data indicate a complex role of uromodulin in kidney homeostasis. Apart from the urinary tract, the compound is also present in the serum. A number of targets were identified, including T lymphocytes [7], transient receptor potential melastatin 2 (TRPM2) [8], and mononuclear phagocytes [9,10]. Therefore, uromodulin is considered an immunoregulatory protein of kidney origin. Uromodulin levels in serum are approximately 100 times lower than those in urine [6]. Among patients with kidney failure, serum uromodulin is often very low or even undetectable [11]. Thus, serum uromodulin levels have been proposed as a marker of tubular mass and function and as a more specific indicator of kidney damage than standard laboratory parameters [11]. Interestingly, serum, but not urinary, uromodulin levels were associated with a lower incidence of kidney events in patients with CKD [12]. Additionally, higher serum uromodulin concentration has been associated with a lower risk of major adverse cardiovascular events and mortality in the CKD population [13]. This correlation was not observed among peritoneal dialysis patients [14]. Nevertheless, the role of serum uromodulin in the progression of kidney disease, treatment response, and overall outcomes among patients receiving KRT remains poorly recognized.

The tryptophan-kynurenine (Trp-KYN) pathway is a major route of Trp degradation. It yields various biologically active products and plays a multifaceted role in kidney disorders. The rate-limiting step is the conversion of Trp to KYN by tryptophan-2,3-dioxygenase (TDO) in the liver or by indoleamine-2,3-dioxygenase (IDO) in peripheral tissues. It is highly dependent on immune system activity, and its alterations have been implicated in various disorders with an immune background [15]. Downstream Trp degradation products, kynurenic acid (KYNA), formed from KYN by kynurenine aminotransferases (KATs), and 3-hydroxykynurenine (3-OHKYN), produced from KYN by kynurenine-3-monooxygenase (KMO), contribute to the pathogenesis of kidney and related disorders [16]. Although controversies exist regarding whether the impact of Trp-KYN pathway metabolites on kidney damage is mediated by impaired filtration and resulting toxin accumulation, some evidence suggests a direct cellular-damaging effect of kynurenines on kidney function [17,18]. Trp-KYN pathway metabolites are predominantly removed from the body through tubular transporters [19]. Consequently, the preservation of RKF appears vital to reducing the incidence of kidney-specific and overall mortality and morbidity. In CKD patients with an estimated glomerular filtration rate (eGFR) of 30–60 mL/min/1.73 m^2^, serum uromodulin concentration was associated with the level of Trp derivative, C-glycosyl Trp, considered a uremic toxin and related to CKD progression [12]. However, the relationship between serum uromodulin levels and other Trp-KYN pathway metabolites in dialyzed patients has not been explored to date.

This preliminary study explores the hypothesis that serum uromodulin levels correlate with Trp-KYN metabolites, potentially revealing new pathophysiological pathways in patients undergoing KRT. Given the link between serum uromodulin, Trp-KYN metabolites, and tubular function, we examined their correlation in KRT patients. Furthermore, we assessed how various clinical and dialysis parameters influence serum uromodulin levels.

## 2. Materials and Methods

### 2.1. Study Group

The study cohort included 64 ambulatory patients with kidney failure from a single center. Study participants were undergoing either bicarbonate HD (*n* = 40) or online post-dilution HDF (*n* = 24) three times per week. Exclusion criteria were: age < 18 years, active neoplastic process, infection or autoimmune disease, hospitalization within last 3 months, transfusion within last 3 months, intake of drugs known to affect the Trp-KYN pathway, pregnancy or breast feeding, presence of severe comorbidities with expected short survival, dialysis in a single needle mode or using non-tunneled catheter, any physical or mental condition compromising the ability to provide informed consent or adhere to study requirements. HD procedures were conducted using Fresenius 4008 monitors (Fresenius Medical Care, Bad Homburg, Germany), whereas HDF was performed with Fresenius 5008 monitors (Fresenius Medical Care, Bad Homburg, Germany). The ultrafiltration (UF) rate was set individually based on the patient’s interdialytic weight gain. All previously prescribed drugs were continued throughout the study without any modifications. Technical dialysis parameters, patients’ demographic and clinical data were collected from medical documentation. RKF, defined as urinary output > 200 mL/day, was assessed based on home measurements performed by patients. Single pool Kt/V was established based on the Daugirdas equation [20]. Essential laboratory parameters were evaluated in every patient before dialysis. All participants were regularly informed about the recommended dietary guidelines for HD patients.

This study received approval from the Bioethical Committee at the Medical University, Lublin, Poland (number KE-0024/6/01/2025), and was performed in accordance with the Declaration of Helsinki. All participants received detailed information about the aim of this study, were allowed sufficient time to review the study protocol and provided written informed consent prior to inclusion.

### 2.2. Sample Collection

Blood samples from each patient were obtained during the midweek dialysis session, at a similar time of month to ensure comparable results between groups with different HD schedules. The levels of tested metabolites were measured in blood samples drawn from the arterial blood line immediately before and after the end of each dialysis session, following a 30 s period of blood flow at 50 mL/min, after which the dialysate flow was stopped. Afterwards, blood samples were centrifuged for 10 min (2700 rpm). For the analysis of Trp-KYN pathway metabolites, plasma samples were acidified with 10% trichloroacetic acid (500 μL added for each 500 μL of plasma) and centrifuged for 10 min (12,000 rpm). Serum samples for immunoenzymatic analyses were centrifuged for 10 min (2700 rpm). The resulting supernatants were maintained at −72 °C until further experimental procedures.

### 2.3. Quantification of Trp, KYN and KYNA

The levels of serum Trp, KYN, and KYNA were assessed through ultra-high-pressure liquid chromatography (UHPLC) (Waters Acquity UHPLC system; Waters C18 analytical column, Milford, MA, USA), as previously presented [21]. In short, the mobile phase containing 20 mM sodium acetate, 3 mM zinc acetate, and 7% acetonitrile was run with a flow rate of 0.1 mL/min. All analyses were performed with an ultraviolet variable wavelength detector (at 250 nm for Trp and at 365 nm for KYN), and a fluorescence detector (344 nm excitation and 398 nm emission for KYNA). The HPLC column (HR-80; 3 µm; C18 reverse-phase column) was perfused at 0.6 mL/min with a mobile phase made from 2% acetonitrile, 0.9% triethylamine, 0.59% phosphoric acid, 0.27 mM sodium ethylenediaminetetraacetic acid (EDTA) and 8.9 mM heptane sulfonic acid. A standard calibration curve was prepared using external standards (containing 0.2, 0.4, 0.6, 0.8, and 1 pmol of KYNA, respectively), with a linearity of >0.999. All standard substances, L-Trp (catalogue number T8941), L-KYN (sulfate salt) (catalogue number K3750), and KYNA (catalogue number K3375) were purchased from Sigma Aldrich (St. Louis, MO, USA). Reagents necessary for HPLC were obtained from J.T.Baker (Avantor, Center Valley, PA, USA). HPLC data were analyzed through Empower 3 software (Waters corporation, Milford, MA, USA, version 7.21.00.00).

### 2.4. Quantification of 3-OHKYN

Serum 3-OHKYN concentration was quantified using an electrochemical detector (Thermo Scientific Dionex UltiMate 3000 ECD-3000RS, ThermoFisher Scientific, Waltham, MA, USA) coupled to an analytical cell, with the oxidation voltage set to 0.6 V, as described by Heyes and Quearry [22]. Waters Spherisorb S3 ODS2 150 × 2.1 mm column (Milford, MA, USA) was perfused with a mobile phase (2% acetonitrile, 0.9% triethylamine, 0.59% phosphoric acid, 0.27 mM sodium EDTA, and 8.9 mM heptane sulfonic acid), with a 0.3 mL/min flow. Necessary HPLC reagents used for 3-OHKYN experiments were purchased from J.T. Baker (Avantor, Center Valley, PA, USA), whereas 3-OH-DL-KYN standard (catalogue number H1771) was obtained from Sigma-Aldrich (St. Louis, MO, USA). Chromeleon software (version 7.2.6, Thermo Fisher Scientific, Waltham, MA, USA) was used to collect and interpret 3-OHKYN data.

### 2.5. Quantification of Uromodulin

Commercially available enzyme-linked immunosorbent assay (ELISA) (catalogue number EQ 6821-9601) was obtained from Euroimmun (Lübeck, Germany) to analyze serum uromodulin concentration in all dialyzed patients. Testing was performed according to the manufacturer’s instructions and without any modifications. Since testing was performed on a validated diagnostic test, each sample was analyzed once. Intra-assay coefficient of variation in the uromodulin test was <10%, whereas the value of the inter-assay coefficient of variation was <12%. The sensitivity of the assay was 2 ng/mL. Patients with serum uromodulin levels below this limit were categorized as participants with undetectable serum uromodulin.

### 2.6. Data Analysis

Removal ratios (RRs) of all analyzed substances were assessed through the Bergström and Wehle equation [23]. Post-dialysis concentration (Cpost) correction for hemoconcentration was made according to the following formula:

$$corr\;Cpost=\frac{Cpost}{\left[1+(\frac{Bw\;pre\;-\;Bw\;post}{0.2\;Bw\;post}\right)}$$

corr Cpost: corrected post-dialysis concentration; Bw pre: pre-dialysis body weight; Bw post: post-dialysis body weight.

The RRs of tested metabolites were established separately for each patient.

To assess the activity of selected KYN pathway enzymes, the following ratios were used—KYN/Trp, KYNA/KYN, and 3-OHKYN/KYN—which reflected the activity of IDO, KAT, and KMO, respectively.

The Shapiro–Wilk test was performed to assess the normality of the variables’ distributions. Due to the non-normal distribution of the data obtained, nonparametric tests were performed in subsequent steps. Continuous data were presented as medians (interquartile ranges [IQRs]), whereas categorical data were expressed as percentages. The comparison of continuous variables among two groups was assessed by the Mann–Whitney U test and analysis of the categorical data was performed with the use of the chi-square test. Spearman’s rank correlation test was performed to assess the association between the continuous variables. Statistical analyses were conducted using STATISTICA 13.3 and GraphPad Prism 6. A *p*-value < 0.05 was considered statistically significant.

## 3. Results

### 3.1. Study Participants

Baseline clinical characteristics of dialyzed patients are shown in Table 1. Among 64 patients enrolled in this study, 32 were male, with a median age of 65 years. The median dialysis vintage was 40.5 months, with the median UF volume of 2.65 L. The median Kt/V was 1.5. A total of 24 patients were treated with HDF, whereas the remaining patients were treated by HD. Diabetes mellitus was reported in 17 patients, hypertension in 55 and heart failure in 21. In total, 35 dialyzed patients had RKF.

Uromodulin serum concentration was determined in 30 dialyzed patients from 64 enrolled in this study. Compared to patients without measurable serum uromodulin concentration, patients with detectable uromodulin had significantly shorter dialysis vintage, more often had preserved RKF, higher diabetes mellitus prevalence, and higher serum pre-dialysis phosphorus concentrations, and used loop diuretics more often (Table 1).

### 3.2. Unadjusted Correlation Between Serum Uromodulin Concentration and Clinical Parameters in Dialyzed Patients

After analyzing clinical parameters, a significant positive correlation between pre-dialysis serum uromodulin concentration and male sex (Rho = 0.4779, *p* < 0.001), diabetes mellitus (Rho = 0.2700, *p* = 0.0309), hypertension (Rho = 0.2572, *p* = 0.0401), RKF (Rho = 0.4809, *p* < 0.001), pre-dialysis phosphorus level (Rho = 0.2756, *p* = 0.0274) and loop diuretics use (Rho = 0.5015, *p* < 0.001) was found (Table 2). On the other hand, serum uromodulin level correlated negatively with dialysis vintage (Rho = −0.4787, *p* < 0.001) and Kt/V value (Rho = −0.2497, *p* = 0.0465). No significant correlations were observed between other clinical variables and pre-dialysis serum uromodulin concentration. The data presented were unadjusted for RKF, obtained on a small sample size after multiple comparisons and the observed correlations should be considered as exploratory findings.

### 3.3. Uromodulin Removal in the Study Group

**Figure 1:**
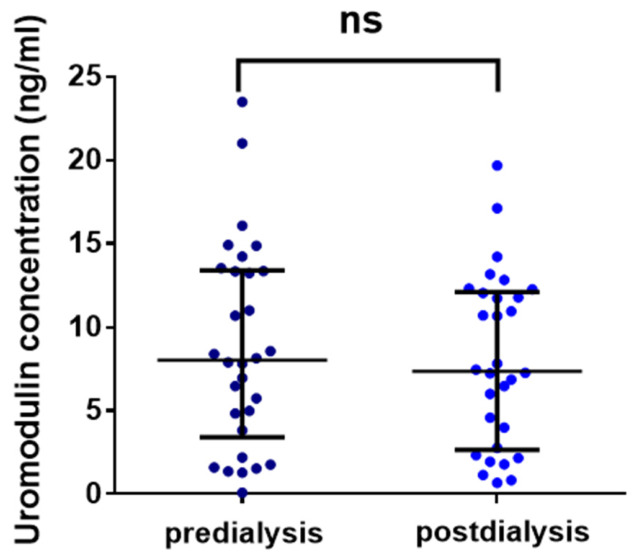
Pre-dialysis and post-dialysis concentration of uromodulin in dialyzed patients (*n* = 30). Mann–Whitney U test. Data are shown as median with interquartile range.

Pre-dialysis uromodulin concentration in dialyzed patients was 8.01 ng/mL [3.81–13.34]. Dialysis did not significantly change uromodulin’s concentration, with a post-dialysis value of 7.36 ng/mL [2.76–12.03], *p* = 0.58 (Figure 1).

RR for uromodulin in dialyzed patients was 5.87% [−4.38–19.69].

**Figure 2:**
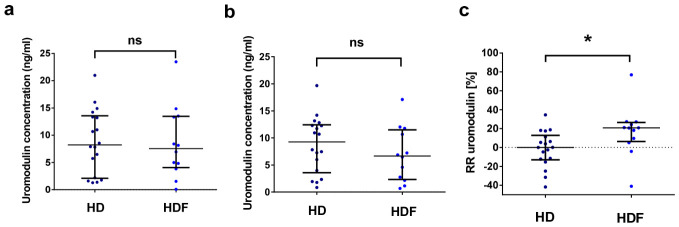
Pre-dialysis (**a**) and post-dialysis (**b**) uromodulin concentrations, and RR for uromodulin (**c**) in patients treated with HD (*n* = 18) and HDF (*n* = 12). Mann–Whitney U test. Data are shown as median with interquartile range. Dotted line separates negative from positive values. * *p* < 0.05.

Among patients with detectable serum uromodulin levels, 18 received HD and 12 received HDF. Compared to the HD group, in HDF-treated patients, pre-dialysis (7.54 ng/mL vs. 8.22 ng/mL, *p* = 0.80) and post-dialysis serum uromodulin levels were not significantly different (6.66 ng/mL vs. 9.25 ng/mL, *p* = 0.26) (Figure 2), whereas RR for uromodulin was higher compared to the HD cohort (19.41% vs. 0.09%, *p* = 0.01).

### 3.4. Trp Metabolites Removal in Dialyzed Patients in Accordance with the Uromodulin Detection

In dialyzed patients, the median pre-dialysis Trp concentration was 8.44 µmol/L (Table 3). Patients with detectable serum uromodulin had significantly higher pre-dialysis Trp levels than patients without detectable serum uromodulin (10.15 µmol/L vs. 7.69 µmol/L, *p* = 0.004).

Similarly, higher post-dialysis Trp concentration was observed in patients with an established serum uromodulin level than in those without detectable uromodulin (9.85 µmol/L vs. 7.92 µmol/L, *p* = 0.025). Remaining pre- and post-dialysis Trp metabolite concentrations and their respective ratios did not differ significantly between patients with and without measurable serum uromodulin.

### 3.5. Unadjusted Correlation Between Serum Uromodulin Concentration and Tested Trp Metabolite Level

When analyzing Trp metabolite levels, a significant positive correlation was found between pre-dialysis uromodulin concentration and pre-dialysis Trp level (Rho = 0.4152, *p* < 0.001), whereas a negative correlation was observed between pre-dialysis uromodulin levels and pre-dialysis KYN/Trp ratio (Rho = −0.3250, *p* = 0.008) (Table 4).

Only post-dialysis Trp concentration positively correlated with serum post-dialysis uromodulin level (Rho = 0.3400, *p* = 0.005); other significant correlations were not found between tested substances in both pre-dialysis and post-dialysis settings.

## 4. Discussion

To our knowledge, this is the first study to investigate the relationship between serum uromodulin levels and Trp metabolites in a dialysis population, and it provides a comprehensive analysis of the KYN pathway in ESKD patients.

Notably, serum uromodulin levels were undetectable in a subset of the study population. Patients with detectable uromodulin were characterized by a male predominance, shorter dialysis vintage, and higher serum phosphorus levels. They also demonstrated a higher frequency of diabetes mellitus, loop diuretic use, and preserved RKF. Unadjusted correlation analysis revealed that serum uromodulin levels were positively correlated with hypertension and negatively correlated with Kt/V. The presented data indicate that serum uromodulin level is strongly correlated with RKF. As uromodulin is a marker of tubular function, the results suggest that serum uromodulin is detectable in patients with preserved diuresis, as indicated by the use of loop diuretics. Shorter dialysis vintage and higher serum phosphorus levels in the uromodulin-positive group, along with a negative correlation between serum uromodulin levels and Kt/V, also suggest a reduced impact of dialysis on diuresis; however, elevated phosphorus levels may reflect the metabolic adaptation period for patients with a shorter KRT history.

RKF is one of the main predictors of CKD patients’ outcomes due to the preservation of water excess and the removal of waste products, resulting in reduced inflammation and improved erythropoietin production, which translates into better quality of life and improved survival [24]. Additionally, the secretion of uromodulin into the serum can be another benefit of preserved kidney function, helping control inflammatory conditions associated with ESKD and KRT alone. Beyond interacting with immune cells, uromodulin was named a ‘cytokine trap’ due to co-precipitation with tumor necrosis factor-α (TNF-α) and interleukin-1β (IL-1β) in patients undergoing HD [25]. Protection against systemic oxidative stress, achieved by blocking TRPM2, which is highly expressed on immune cells, or by decreasing the level of 8-hydroxy-2′-deoxyguanosine, a marker of oxidative damage [8], is another effect provided by uromodulin. Since uromodulin deficiency is known to result in a pro-inflammatory milieu and exacerbate oxidative stress, preservation of diuresis should be pivotal in ESKD patients to maintain uromodulin production. Contrary to previous studies, in which females were shown to have higher serum uromodulin levels [26], the present study found that the group with detectable serum uromodulin was predominantly male. However, available observations from the literature were made in healthy individuals, indicating potential sexual dimorphism in uromodulin’s expression. Similar discrepancies were observed in relation to diabetes mellitus, as lower serum uromodulin has been associated with impaired glucose metabolism in patients with CKD [27]. Interestingly, the positive correlation between the presence of hypertension and serum uromodulin concentration remains noteworthy. Based on preclinical study results, uromodulin was shown to increase distal sodium reabsorption by activating the Na^+^-K^+^-2Cl^−^ cotransporter type 2 in the TAL, thereby contributing to sodium-sensitive blood pressure control [28]. Uromodulin knockout mice were shown to have significantly lower blood pressure than wild-type animals, suggesting a role for uromodulin in blood pressure regulation [29]. This contrasts with data on serum uromodulin, different from its tissue variant, which was associated with lower blood pressure values [7]. Additionally, in a study by Chen et al., patients with more intensive blood pressure control had greater declines in serum uromodulin levels, with these differences attributable to factors beyond antihypertensive drug use and not associated with CKD progression [30]. While our findings indicate that serum uromodulin levels are closely associated with RKF and may correlate with specific clinical parameters, the small sample size and limited subgroup sizes constrain our conclusions. These results warrant validation in larger, more diverse cohorts with appropriate adjustment for RKF.

Patients with detectable serum uromodulin had higher pre- and post-dialysis Trp levels, whereas other Trp metabolites and their RRs did not differ significantly. Additionally, pre-dialysis serum uromodulin concentration correlated positively with pre-dialysis Trp level and negatively with the pre-dialysis KYN/Trp ratio, whereas other Trp-KYN pathway metabolites and their respective values did not. RKF likely plays a significant role in shaping these results and should be considered a major confounding factor. The activation of the Trp-KYN pathway in patients undergoing KRT is well documented and is associated with subsequent oxidative stress and cellular injury. The relationship between higher Trp metabolite concentrations and poor cardiovascular prognosis is largely mediated by endothelial dysfunction [31] and impaired hemostasis [32]. Notably, IDO stimulation (expressed as the KYN/Trp ratio) appears particularly pronounced in HD patients and correlates significantly with oxidative stress markers [33]. In this regard, the presence of detectable serum uromodulin may indicate attenuated immunological activation, leading to diminished activity within the Trp-KYN pathway. This is consistent with our findings, in which pre-dialysis serum uromodulin correlated only with Trp concentration and the KYN/Trp ratio, but not with other metabolites also subject to tubular transport.

An ongoing debate about the advantages of HDF over HD in ESKD patients’ survival appears to provide more evidence on quality-of-life, cardiovascular, and mortality improvements in HDF-treated patients [1]. There is a broad consensus that expanded removal of middle molecules and improved cardiovascular stability during HDF procedures seem to have an essential effect on patients’ outcomes, together with a reduction in inflammatory and oxidative stress markers [2]. However, some controversies occur regarding the loss of protein and other essential compounds during HDF procedures. Several studies have shown that HDF can lead to higher albumin and total protein losses regardless of the dialyzer type used [34,35]. We observed a higher RR for uromodulin in the HDF group than in HD patients. However, it seems that this potential decrease in serum uromodulin levels does not lead to long-term adverse outcomes, since pre-dialysis uromodulin concentration did not differ significantly between HD and HDF patients. Although the loss of proteins during HDF is considered most beneficial due to the greater removal of pro-inflammatory cytokines, β2-microglobulin, or indoxyl sulfate [36], the significance of the higher RR for uromodulin should be verified in future studies. Factors such as membrane type, convection volume, treatment time, albumin loss and protein binding can affect presented results and should be evaluated in a larger cohort of patients.

In the current study, serum uromodulin was detectable in only 30 patients, while levels in the remaining participants fell below the assay’s limit of detection. This should be considered a technical limitation of the current uromodulin measurement method. The sensitivity of the uromodulin ELISA used to measure serum protein concentration was 2 ng/mL, similar to that reported in previous studies [13,14]. Additionally, the pre-dialysis uromodulin level in our study was 8.01 ng/mL, similar to the 9.2 ng/mL observed in peritoneal dialysis patients [14]. As most studies report undetectable uromodulin levels in patients on KRT, this study provides insight into uromodulin’s metabolism, although it is limited by the analytical method used.

## 5. Limitations

The findings of this study should be interpreted considering certain limitations.

### 5.1. Sample, Setting, Dialysis

First, the study was conducted at a single dialysis center with a relatively small cohort. Therefore, larger, multi-center studies are required to confirm our findings and ensure their generalizability. Furthermore, dialysis-related factors, including membrane type, convection volume, procedure duration, albumin loss, and protein binding, should be considered in future analyses. Lack of adjustment for RKF limits statistical robustness; thus, it should be considered in future studies.

### 5.2. Assessment of RKF and Urine Output

Second, the assessment of RKF was limited. RKF was estimated based on self-reported home measurements of urine output by non-hospitalized patients, which may introduce variability and affect the interpretation of daily volumes. Direct measurement of urine output was not performed, so potential discrepancies in volume assessment cannot be entirely ruled out.

### 5.3. Temporal and Dietary Factors

Third, while midweek metabolite evaluation is a standard clinical procedure, intermittent dialysis (HD or HDF) may not fully capture fluctuations in Trp and uromodulin throughout the interdialytic period. Furthermore, although patients received dietary counseling, their food intake was not strictly standardized. Consequently, the influence of varying dietary Trp levels on our results cannot be unequivocally excluded.

### 5.4. Microbiota and Biological Variability

Fourth, we did not examine the intestinal microbiota composition. Since the production of Trp metabolites can be influenced by gut flora, this variability might have impacted our findings, even though no patients reported gastrointestinal symptoms during the study.

### 5.5. Biomarker Specificity and Blood Sampling

Finally, our analysis focused exclusively on serum uromodulin, as it is considered a more sensitive predictor of CKD than urinary levels—the latter being difficult to obtain in patients with minimal or no RKF. However, no tissue-level concentrations were evaluated. Additionally, potential interference from other uremic toxins with our analytical methods should be considered.

Although blood sample processing may affect the evaluation of uromodulin levels, serum protein is much more stable than urinary uromodulin [37,38]. Polymerization of urinary uromodulin is another factor affecting antibody-mediated techniques’ results; thus, serum protein level establishment remains a more reliable method of uromodulin level analysis [39].

## 6. Conclusions

Our study highlights the differences in Trp metabolism in patients with and without detectable serum uromodulin levels. Uromodulin’s concentration in the serum may correlate with KYN pathway metabolite levels in dialyzed patients. The potential impact of HDF on uromodulin loss in dialyzed patients needs further evaluation. Future studies are encouraged to verify the significance of the presented results in longitudinal outcomes of dialyzed patients.

## Figures and Tables

**Table 1:** Baseline characteristics of dialyzed patients. Continuous data are shown as medians with interquartile ranges, whereas categorical data are presented as percentages. Mann–Whitney U test or chi-square test, respectively. Statistically significant data are shown in bold. Abbreviations: 3-OHKYN, 3-hydroxykynurenine; HD, hemodialysis; HDF, hemodiafiltration; KYN, kynurenine; KYNA, kynurenic acid; RKF, residual kidney function; Trp, tryptophan; UF, ultrafiltration; URR, urea reduction ratio.

Variable	All Participants	Uromodulin Negative	Uromodulin Positive	*p*
Age (years)	65 [55–73]	68 [56–73]	64 [51–71]	0.329
Male, *n* (%)	32 (50)	9 (14)	23 (36)	**<0.001**
Dialysis vintage (months)	40.5 [12–71]	58.5 [27–103]	15 [11–59]	**<0.001**
HD length (h/week)	12 [10.75–12]	11.5 [11–12]	12 [10.5–12]	0.505
Body mass (kg)	70 [60.75–81.75]	66.25 [57–78.5]	75.75 [62.5–86]	0.058
UF volume (L)	2.65 [1.9–3.2]	2.65 [2.0–3.2]	2.65 [1.8–3.0]	0.549
HDF, *n* (%)	24 (38)	12 (19)	12 (19)	0.571
Kt/V	1.5 [1.33–1.69]	1.55 [1.42–1.73]	1.41 [1.27–1.64]	0.092
URR (%)	72.5 [67–75]	73 [70–75]	69 [66–75]	0.102
Diabetes mellitus, *n* (%)	17 (26.5)	5 (8)	12 (18.5)	**0.028**
Hypertension, *n* (%)	55 (86)	26 (41)	29 (45)	0.334
Heart failure, *n* (%)	21 (33)	11 (17)	10 (16)	0.500
RKF, *n* (%)	35 (54.5)	11 (17)	24 (37.5)	**0.001**
Pre-dialysis systolic blood pressure (mmHg)	139.5 [129–156.5]	136.5 [126–153]	142.5 [134–163]	0.146
Pre-dialysis diastolic blood pressure (mmHg)	72.5 [68–80]	71.5 [65–77]	73 [69–80]	0.322
Hemoglobin (g/dL)	11.4 [10.6–12.15]	11.5 [10.2–12.3]	11.35 [10.7–12]	0.558
Pre-dialysis urea (mg/dL)	111.4 [94.1–133.2]	107.4 [95.1–126.2]	116.4 [88.1–140.9]	0.396
Pre-dialysis potassium (mmol/L)	5.3 [4.8–5.6]	5.4 [4.9–5.6]	5.1 [4.7–5.6]	0.239
Pre-dialysis calcium (mg/dL)	8.95 [8.55–9.25]	8.75 [8.5–9.2]	9.0 [8.7–9.3]	0.141
Pre-dialysis phosphorus (mg/dL)	5.1 [4.25–6.3]	4.65 [4.29–5.9]	5.5 [5.0–6.9]	**0.015**
Parathyroid hormone (pg/mL)	270 [173.5–425.5]	300.5 [164–511]	260 [179–401]	0.632
B-type natriuretic peptide (pg/mL)	214.5 [105.5–551]	266 [145–615]	164 [94–324]	0.087
Uric acid (mg/dL)	5.3 [4.8–6.0]	5.35 [4.8–6.0]	5.3 [4.8–6.2]	0.935
C-reactive protein (mg/dL)	5.45 [2.7–10.05]	5.75 [2.4–9]	5.2 [3.4–10.4]	0.632
Loop diuretics (%)	40 (62.5)	14 (22)	26 (40.5)	**0.004**

**Table 2:** Unadjusted correlation between pre-dialysis concentration of uromodulin and selected clinical variables in dialyzed patients. Spearman’s correlation test. Data with statistical significance are highlighted in bold. Abbreviations: HD, hemodialysis; HDF, hemodiafiltration; RKF, residual kidney function; UF, ultrafiltration; URR, urea reduction ratio.

Variable	Pre-Dialysis Uromodulin	
	Rho	*p*-Value
Age	−0.0586	0.6453
Male sex	0.4779	**<0.001**
Dialysis vintage	−0.4787	**<0.001**
HD length	0.0360	0.7770
Body mass	0.1872	0.1385
UF volume	−0.2033	0.1071
HDF	0.0341	0.7890
Kt/V	−0.2497	**0.0465**
URR	−0.2295	0.0680
Diabetes mellitus	0.2700	**0.0309**
Hypertension	0.2572	**0.0401**
Heart failure	0.0156	0.9024
RKF	0.4809	**<0.001**
Pre-dialysis systolic blood pressure	0.1981	0.1164
Pre-dialysis diastolic blood pressure	0.1797	0.1551
Hemoglobin	−0.1166	0.3587
Pre-dialysis urea	0.0697	0.5841
Pre-dialysis potassium	−0.1176	0.3543
Pre-dialysis calcium	0.1642	0.1946
Pre-dialysis phosphorus	0.2756	**0.0274**
Parathyroid hormone	−0.0998	0.4325
B-type natriuretic peptide	−0.2000	0.1129
Uric acid	0.0242	0.8488
C-reactive protein	0.0165	0.8966
Loop diuretics	0.5015	**<0.001**

**Table 3:** Unadjusted pre- and post-dialysis concentrations of the examined metabolites, along with related ratios indicating KYN pathway enzyme activity and RRs for each metabolite in patients with and without detected serum uromodulin. Data are shown as medians with interquartile range. Mann–Whitney U test. Statistically significant data are shown in bold. Abbreviations: 3-OHKYN, 3-hydroxykynurenine; KYN, kynurenine; KYNA, kynurenic acid; Trp, tryptophan.

Variable	All	Uromodulin Negative	Uromodulin Positive	*p*
Pre-dialysis Trp (µmol/L)	8.44 [5.91–11.70]	7.69 [5.18–9.62]	10.15 [7.56–14.82]	**0.004**
Pre-dialysis KYN (µmol/L)	1.94 [1.35–2.63]	1.99 [1.37–2.39]	1.93 [1.30–2.74]	0.882
Pre-dialysis KYNA (nmol/L)	415.15 [288.55–612.30]	469.55 [269.80–667.90]	385.55 [307.30–533.50]	0.233
Pre-dialysis 3-OHKYN (nmol/L)	743.65 [520.80–976.65]	754.50 [396–1259.7]	743.65 [534.20–886]	0.681
Pre-dialysis KYN/Trp	0.20 [0.12–0.37]	0.26 [0.15–0.56]	0.17 [0.11–0.33]	0.056
Pre-dialysis KYNA/KYN	220.04 [125.50–354]	265.72 [131.90–371.73]	195.89 [109.39–290.34]	0.233
Pre-dialysis 3-OHKYN/KYN	316.71 [217.73–810.34]	354.01 [204.86–1179.57]	310.86 [262.19–462.87]	0.782
Post-dialysis Trp (µmol/L)	8.46 [6.25–11.90]	7.92 [5.49–10.09]	9.85 [6.95–12.67]	**0.025**
Post-dialysis KYN (µmol/L)	0.73 [0.42–1.00]	0.59 [0.38–0.98]	0.82 [0.48–1.13]	0.385
Post-dialysis KYNA (nmol/L)	226.03 [161.23–364.29]	233.87 [172.85–411.80]	187.12 [150.39–273.31]	0.120
Post-dialysis 3-OHKYN (nmol/L)	223.67 [142.37–306.71]	220.41 [124.65–285.16]	226.26 [144.77–322.39]	0.908
Post-dialysis KYN/Trp	0.08 [0.05–0.10]	0.08 [0.06–0.10]	0.07 [0.05–0.11]	0.239
Post-dialysis KYNA/KYN	309.35 [171.12–699.19]	376.01 [209.84–834.75]	244.82 [148.31–527.16]	0.094
Post-dialysis 3-OHKYN/KYN	288.18 [151.63–558.16]	326.35 [107.61–1017.54]	283.40 [214.49–358.73]	0.941
RR Trp (%)	6.06 [−15.33–22.89]	1.71 [−19.35–21.51]	12.63 [−4.44–29.41]	0.088
RR KYN (%)	61.00 [42.04–72.97]	63.12 [42.88–81.62]	59.48 [41.20–72.61]	0.471
RR KYNA (%)	45.42 [37.98–52.05]	45.16 [35.22–52.92]	46.46 [38.20–52.03]	0.585
RR 3-OHKYN (%)	68.40 [60.93–80.42]	70.59 [62.46–81.69]	66.97 [56.29–74.81]	0.233

**Table 4:** Unadjusted correlation between uromodulin and selected Trp metabolites concentration in dialyzed patients. Spearman’s correlation test. Data with statistical significance are highlighted in bold. Abbreviations: 3-OHKYN, 3-hydroxykynurenine; KYN, kynurenine; KYNA, kynurenic acid; RR, reduction ratio; Trp, tryptophan.

Variable	Pre-Dialysis Uromodulin		Variable	Post-Dialysis Uromodulin	
	Rho	*p*-Value		Rho	*p*-Value
Pre-dialysis Trp	0.4152	**<0.001**	Post-dialysis Trp	0.3400	**0.005**
Pre-dialysis KYN	−0.0602	0.635	Post-dialysis KYN	0.0862	0.498
Pre-dialysis KYNA	−0.1868	0.139	Post-dialysis KYNA	−0.1949	0.122
Pre-dialysis 3-OHKYN	−0.0539	0.672	Post-dialysis 3-OHKYN	0.0142	0.911
Pre-dialysis KYN/Trp	−0.3250	**0.008**	Post-dialysis KYN/Trp	−0.1997	0.113
Pre-dialysis KYNA/KYN	−0.0872	0.493	Post-dialysis KYNA/KYN	−0.2035	0.106
Pre-dialysis 3-OHKYN/KYN	0.0258	0.839	Post-dialysis 3-OHKYN/KYN	0.0036	0.976
RR Trp	0.1483	0.262	RR Trp	0.1235	0.351
RR KYN	−0.1854	0.152	RR KYN	−0.1889	0.144
RR KYNA	0.0009	0.994	RR KYNA	−0.0017	0.989
RR 3-OHKYN	−0.1296	0.307	RR 3-OHKYN	−0.1386	0.274

## Data Availability

The datasets generated and analyzed during the current study are available from the corresponding author on reasonable request.

## Abbreviations

The following abbreviations are used in this manuscript:

3-OHKYN	3-hydroxykynurenine
Bw pre	Pre-dialysis body weight
Bw post	Post-dialysis body weight
CKD	Chronic kidney disease
Corr Cpost	Corrected post-dialysis concentration
Cpost	Post-dialysis concentration
EDTA	Ethylenediaminetetraacetic acid
eGFR	Estimated glomerular filtration rate
ELISA	Enzyme-linked immunosorbent assay
ESKD	End-stage kidney disease
HD	Hemodialysis
HDF	Hemodiafiltration
HPLC	High-performance liquid chromatography
IDO	Indoleamine-2,3-dioxygenase
IL-1β	Interleukin-1β
IQR	Interquartile range
KAT	Kynurenine aminotransferase
KMO	Kynurenine-3-monooxygenase
KRT	Kidney replacement therapy
KYN	Kynurenine
KYNA	Kynurenic acid
RKF	Residual kidney function
RR	Reduction ratio
TAL	Thick ascending loop of Henle
TDO	Tryptophan-2,3-dioxygenase
TNF-α	Tumor necrosis factor-α
Trp	Tryptophan
Trp-KYN	Tryptophan-kynurenine
TRPM2	Transient receptor potential melastatin 2
UF	Ultrafiltration
UHPLC	Ultra-high-pressure liquid chromatography
URR	Urea reduction ratio
